# ZO-1 Knockout by TALEN-Mediated Gene Targeting in MDCK Cells: Involvement of ZO-1 in the Regulation of Cytoskeleton and Cell Shape

**DOI:** 10.1371/journal.pone.0104994

**Published:** 2014-08-26

**Authors:** Shinsaku Tokuda, Tomohito Higashi, Mikio Furuse

**Affiliations:** 1 Division of Cell Biology, Department of Physiology and Cell Biology, Kobe University Graduate School of Medicine, Kobe, Japan; 2 Division of Cerebral Structure, National Institute for Physiological Sciences, Okazaki, Japan; NCMLS, Radboud University Nijmegen Medical Center, Netherlands

## Abstract

ZO-1, ZO-2 and ZO-3 are tight junction-associated scaffold proteins that bind to transmembrane proteins of tight junctions and the underlying cytoskeleton. ZO-1 is involved in the regulation of cytoskeletal organization, but its detailed molecular mechanism is less well understood. Gene knockout is an ideal method to investigate the functions of proteins that might have redundant functions such as ZO proteins, when compared with methods such as RNA interference-mediated suppression of gene expression. In this study we applied transcription activator-like effector nucleases (TALENs), a recently developed genome editing method for gene knockout, and established ZO-1 knockout clones in Madin-Darby canine kidney (MDCK) cells. ZO-1 knockout induced striking changes in myosin organization at cell–cell contacts and disrupted the localization of tight junction proteins; these findings were previously unseen in studies of ZO-1 knockdown by RNA interference. Rescue experiments revealed that trace ZO-1 expression reversed these changes while excessive ZO-1 expression induced an intensive zigzag shape of cell–cell junctions. These results suggest a role for ZO-1 in the regulation of cytoskeleton and shape of cell–cell junctions in MDCK cells and indicate the advantage of knockout analysis in cultured cells.

## Introduction

In multicellular organisms, epithelia act as a barrier to the external environment. Epithelial cells adhere to each other through complexes that form junctions between the cells, and the tight junction (TJ) is located in the most apical part of the complexes [1]. TJs regulate movements of several substances through the paracellular pathway to maintain homeostasis in the internal environment required for proper organ function [2], [3].

The zonula occludens-1 (ZO-1) was the first TJ protein to be identified [4]. ZO-1 is a multi-domain scaffolding protein that belongs to the TJ MAGUK (membrane-associated guanylate kinase-like homologs) family that also contains ZO-2 and ZO-3 [5]–[8]. ZO proteins bind to the transmembrane proteins of TJs such as occludin and claudins [9], [10] as well as F-actin and many other regulatory components of the cytoskeleton, thus cross-linking TJ proteins to the underlying cortical cytoskeleton [11], [12]. Therefore, ZO proteins are likely to be important for the regulation of the cytoskeleton at TJs, and consistent with this idea, the suppression of ZO-1 expression by RNA interference (knockdown) in Madin-Darby canine kidney (MDCK) II cells was reported to change the organization of the perijunctional cytoskeleton and the shape of cell–cell junctions from tortuous to linear [13]. However, two other studies did not note these changes in ZO-1 knockdown MDCK II cells [14], [15]. Because RNAi-mediated knockdown is not complete and only reduces gene function, one explanation for the differences observed between these studies might be differences in the levels of remaining ZO-1 expression [13].

The complete elimination of gene function through changes in the genetic code (knockout) is an ideal method for analysis of genes, especially of functionally redundant proteins such as ZO proteins [16]. However, there have been few reports of knockout analysis by homologous recombination in cultured cells because of the low efficiency of recombination and the need for repetitive drug selection to disrupt each allele. Furthermore, it is difficult to disrupt genes that have more than two alleles [16], [17]. Recently, genetic engineering has improved by the use of zing-finger nucleases (ZFNs), transcription activator-like effector nucleases (TALENs), and the clustered regularly interspaced short palindromic repeats (CRISPR)/Cas system [18]–[20]. These techniques are being used increasingly to knockout genes in model organisms and cultured cells [21], but to date there have been no reports comparing knockout analysis of target genes using these methods with knockdown analysis in cultured cells. Since TALENs are easy to construct compared with ZFNs, and the CRISPR/Cas system may have a problem with specificity [21], here we chose TALENs to knockout ZO-1 in MDCK II cells.

Transcription activator-like effectors (TALEs) are natural bacterial proteins secreted by *Xanthomonas sp.* which contain tandem repeats of DNA binding domains that recognize specific nucleotides [22]. TALENs are artificial nucleases generated by fusing a *Fok*I DNA cleavage domain to TALEs. Two TALENs that recognize the left and right arms of the target site form a functional *Fok*I dimer and induce DNA double-strand breaks (DSBs) on the target site. Normally, DSBs are repaired by the nonhomologous end-joining pathway (NHEJ), resulting in the introduction of nucleotide mismatches, insertions or deletions and functional gene knockout [19].

In this study, we first constructed TALENs for the knockout of ZO-1. These TALENs effectively knocked out ZO-1 expression in MDCK cells. Then we established ZO-1 knockout clones in MDCK II cells. We found a striking change in myosin organization at cell–cell contacts and a disruption in the localization of TJ proteins in these clones. These changes were reversed by trace ZO-1 expression. In addition, excessive ZO-1 expression induced an intensive zigzag shape of cell–cell junctions. Our results suggest that ZO-1 plays an important role in the regulation of cytoskeleton and cell–cell junction shape in MDCK cells and indicate the importance of knockout analysis in cultured cells.

## Materials and Methods

### Cells, antibodies and reagents

MDCK II cells were provided by Dr. Masayuki Murata. MDCK I cells were obtained from the late Dr. Shoichiro Tsukita (Kyoto University) and were maintained in our laboratory. Cells were grown in DMEM (high glucose) supplemented with 5% fetal bovine serum.

Mouse anti-ZO-1 monoclonal antibody (Ab) (T8/754), rabbit anti-occludin polyclonal antibody and rat anti-occludin monoclonal Ab (MOC37), rabbit anti-claudin-2 polyclonal antibody, and rabbit anti-claudin-4 antibody were characterized as described previously [23]–[26]. Rabbit anti-ZO-2 polyclonal Ab (38–9100), rabbit anti-ZO-3 polyclonal Ab (36–4100), rabbit anti-claudin-1 polyclonal Ab (32–5600), rabbit anti-claudin-3 polyclonal Ab (34–1700), mouse anti-claudin-4 monoclonal Ab (32–9400), and rabbit anti-claudin-7 polyclonal Ab (34–9100) were purchased from Invitrogen. Rabbit anti-FLAG polyclonal Ab (PM020) was purchased from Medical and Biological Laboratories. Rabbit anti-nonmuscle myosin heavy chain II-B (MHC-B) polyclonal Ab (PRB-445P) was purchased from Covance. Mouse anti-phospho-myosin light chain 2 (Ser19) monoclonal Ab (#3675) was purchased from Cell Signaling. Mouse anti-E-cadherin monoclonal Ab (ECCD-2; M108) was purchased from Clontech. Blebbistatin and fluorescein isothiocyanate-dextran (FITC-dextran) were purchased from Sigma-Aldrich.

### Construction of TALENs and establishment of knockout clones

TALENs were constructed following the detailed instruction provided by the TALE Toolbox kit from the Zhang laboratory [27] (Addgene, #1000000019). To establish ZO-1 knockout clones, a pair of TALEN constructs for ZO-1 knockout were cloned into a mammalian expression vector pCAGGS [28] with a neomycin resistance gene and puromycin resistance gene. Cells were seeded in a 6-well plate (Falcon) at a density of 4×10^4^ cells/well and these vectors were transfected into cells 2 h after seeding using Lipofectamine LTX with Plus Reagent (Invitrogen) following the manufacturer's protocol. Then 500 µg/ml G418 and 5 µg/ml puromycin were administered for 4 h on day after transfection. Remaining clones were isolated and screened for ZO-1 depletion by immunocytochemistry.

### cDNA cloning and plasmid construction

cDNA encoding mouse ZO-1 described previously [29] was cloned into pCAGGS with N-terminal 1×FLAG (DYKDDDDK) tag and 2×Strep II (WSHPQFEK) tags or pCAGGS with N-terminal Venus. To establish stably expressing clones, the vectors were transfected into cells and stable clones were selected in standard media supplemented with 500 µg/ml G418.

### DNA sequencing analysis

DNA sequencing was performed using the dideoxy chain termination method with BigDye Terminator version 3.1 Cycle Sequencing Kit (Applied Biosystems) and results were analyzed by the Applied Biosystems 3130 Genetic Analyzers (Applied Biosystems). The chromatograms of sequence results were analyzed using Peak Scanner Software 2 (Applied Biosystems).

### PCR amplification of genomic DNA

Genomic DNA were isolated by the Hot-shot method [30] and subjected to PCR for the amplification of TALEN targeting site in ZO-1 gene (Forward: 5′-AAGGAAGTTCCTGCGTGTAG-3′; Reverse: 5′-TCTGACTTCCTGACATCTGG-3′) and TALEN C-terminal region (Forward: 5′-CTGCGGCACAAATTGAAATA-3′; Reverse: 5′- ATGAGCGGAAATTGATCTCG-3′). PCR products of TALEN targeting site in ZO-1 gene were directly subjected to sequence analysis. For ZO-1 knockout clones 2 and 3, PCR products were cloned into pCAGGS and subjected to sequence analysis.

### Immunocytochemistry

Immunocytochemistry was performed on cells cultured on 12-mm-diameter Transwell filter inserts with a 0.4-µm pore size (Corning, Corning, NY). Cells were plated at a density of 2×10^5^ cells/cm^2^ and cultured for 4 d. Filter inserts were fixed in 1% paraformaldehyde at room temperature for 10 min. In the case of immunostaining for MHC-B, filters were fixed in 100% methanol for 10 min at −20°C. Then filters were permeabilized in a solution of 0.2% (w/v) Triton X-100 (EMD Biosciences) in PBS for 60 min, blocked with 2% bovine serum albumin and incubated with a primary Ab followed by a fluorescence-labeled secondary Ab. Filamentous actin (F-actin) was visualized using alexa fluor 488 phalloidin (Molecular Probes, Invitrogen, A12379). Samples were imaged on a Zeiss LSM700 confocal microscope using a 63× Plan Apo lens. Signal intensity of the acquired images were analyzed using LSM software ZEN 2009 (Zeiss). Contrast adjustment was generated using Adobe Photoshop (ver. 7.0).

### Immunoblotting

For immunoblotting of cell lysates, cells cultured on Transwell filter inserts were scraped into Laemmli SDS sample buffer and boiled for 5 min. The proteins were separated by one-dimensional SDS-PAGE and electrotransferred from the gels to PVDF membranes followed by incubation with primary Abs. Bound Abs were detected using HRP-linked secondary Abs and visualized by enhanced chemiluminescence (ECL Prime Kit; GE Healthcare).

### Quantification of the degree of zigzag of cell–cell junctions (zigzag index)

Stacked confocal images of ZO-3 staining at the level of tight junctions were acquired, saved as TIFF files and opened in ImageJ 1.43u (available at http://rsb.info.nih.gov/ij; developed by Wayne Rasband, National Institutes of Health, Bethesda, MD). Sides of polygonal shapes of cell–cell junctions were determined from the ZO-3 signal, and all sides contained in an area of 840 µm^2^ were manually traced with freehand lines or straight lines (Fig. S1). Five areas were randomly captured for one sample. The ratio of the sum of freehand lines (L_TJ_) to that of straight lines (L_St_) was calculated as L_TJ_/L_St_, and defined as the zigzag index. More than 80 sides were analyzed for one sample, and 3–4 samples were analyzed for each clone.

Statistical comparisons were performed using the Student's *t*-test, and *p*<0.05 was considered statistically significant.

### Time-lapse imaging

MDCK II cells expressing Venus-ZO-1 were cultured on the reverse side of Transwell filter inserts for 3 d, then time-lapse images of fluorescent Venus signal were collected every 30 min for 24 h at 37°C. To adjust the focal point deviation during the time-lapse imaging, we collected time-lapse images at 5 points of z axis, and images at the level of TJs were selected for figures and movie. Time-lapse imaging was performed with Metamorph software (version 7.6, Molecular Devices) equipped with an Olympus IX81 using 60× Plan Apo lens through a cooled charge-coupled device camera (ORCA-ER, Hamamatsu).

### Barrier assays: electrophysiological measurements and tracer flux

Electrophysiological studies were performed as described previously [31]. Cells were plated at a density of 2×10^5^ cells/cm^2^ on Transwell filter inserts, and electrical resistance across the cell monolayer was measured using Millicell-ERS epithelial volt-ohm meter (Millipore) every day for 6 d, and transepithelial electrical resistance (TER) was determined by the subtraction of the resistance of the blank filter. To determine the ion permeability of Na^+^ (*P*_Na_) and Cl^−^ (*P*_Cl_), the dilution potentials and TER of cell monolayers cultured for 6 d were measured with solution A [140 mM NaCl, 5 mM glucose, 5 mM KCl, 1 mM MgCl_2_, 1 mM CaCl_2_ and 10 mM HEPES-NaOH (pH 7.4)] in the apical side and solution B [70 mM NaCl, 130 mM sucrose, 5 mM glucose, 5 mM KCl, 1 mM MgCl_2_, 1 mM CaCl_2_ and 10 mM HEPES-NaOH (pH 7.4)] in the basolateral side at 37°C, and the electrical potentials and resistance of the blank filter under the same condition was subtracted. The stabilities of the transepithelial electrical potential and resistance were confirmed by repetitive measurements for at least 5 minutes. The *P*_Na_/*P*_Cl_ ratio was calculated using the Goldman–Hodgkin–Katz equation. The values for *P*_Na_ and *P*_Cl_ were then calculated from the TER and *P*_Na_/*P*_Cl_ using the Kimizuka–Koketsu equation [32].

For measurements of tracer flux, cell monolayers cultured for 6 d were incubated in solution A with 0.2 mM FITC- dextran in the basolateral side for 1 h, and the solutions in the apical side were collected. The fluorescence of the solutions at 518 nm was measured using a fluorescence spectrophotometer (F-4500; Hitachi High-Tech) with an excitation wavelength of 488 nm, and the amount of FITC–dextran was determined by extrapolation from a standard curve of known fluorescein or FITC–dextran concentrations using linear regression. The permeability of FITC-dextran was defined as (dQ/dt)/AC0 [31].

## Results

### ZO-1 knockout by TALEN-mediated gene targeting changes cell–cell junction shape in MDCK cells

**Figure 1:**
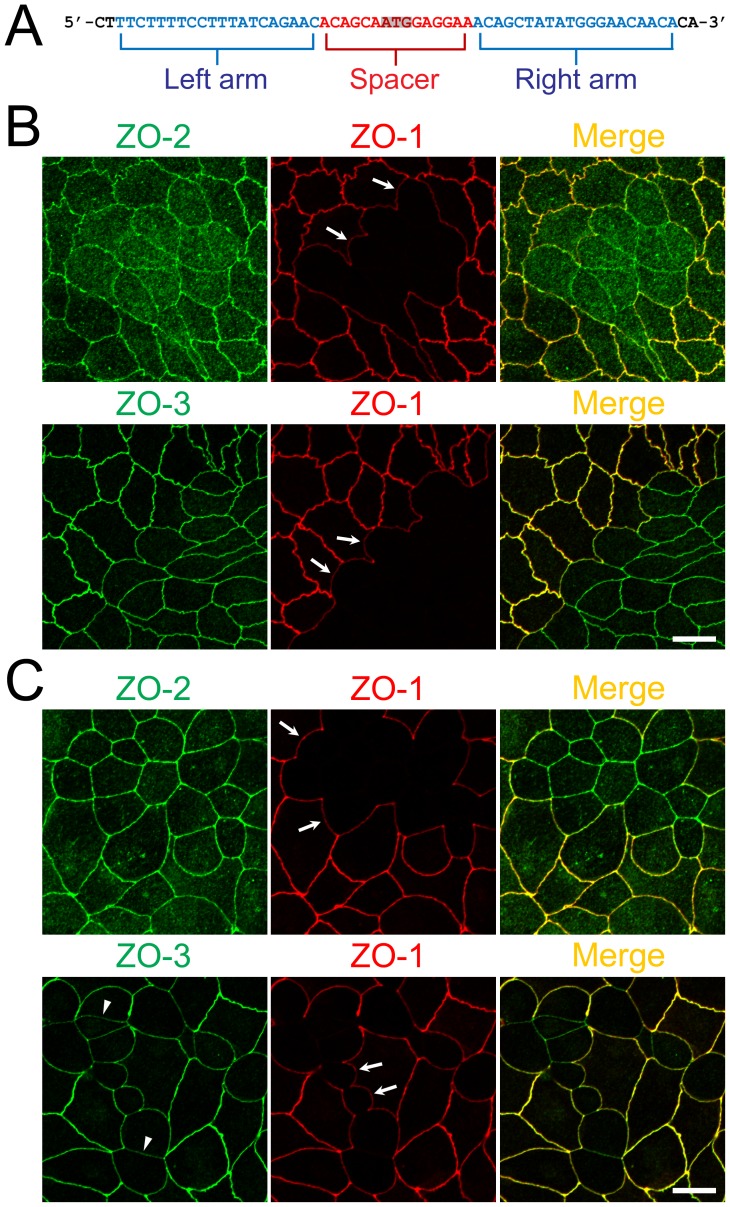
Construction of TALENs and ZO-1 gene knockout in MDCK I and II cells. (A) TALEN binding sites in the ZO-1 gene. The left and right arms of TALEN targeting sites are indicated in blue and the spacer region is indicated in red. The initiation codon within the spacer region is highlighted. (B) Immunofluorescence microscopic analysis of ZO-1, ZO-2 and ZO-3 in MDCK II cells transfected with TALEN constructs for ZO-1 gene knockout. After transfection, cells were subcultured on filter inserts for 4 d before analysis. At the boundary of control and ZO-1 knockout cells, characteristic convex curves of cell–cell junctions are observed (*arrows*). (C) Immunofluorescence microscopic analysis of ZO-1, ZO-2 and ZO-3 in MDCK I cells transfected with TALEN constructs for ZO-1 gene knockout. Similar morphological changes of cell–cell junctions at the boundary of control and ZO-1 knockout cells were observed in MDCK I cells. Staining of ZO-3 was reduced in ZO-1 knockout cells (*arrowheads*). Scale bars, 10 µm.

**Figure 2:**
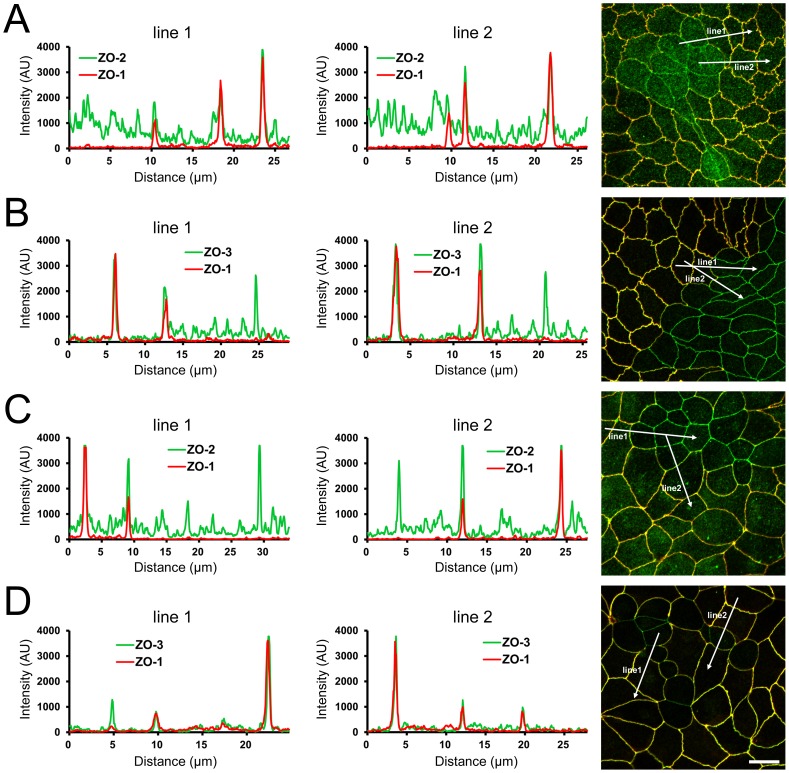
Effects of ZO-1 knockout on the localization of ZO-2 and ZO-3. (A) Effects of ZO-1 knockout on the localization of ZO-2 in MDCK II cells. The images in Figure 1 were used for the analysis. Signal intensity of ZO-2 and ZO-1 on lines shown in confocal microscopic image (*arrows*) were analyzed. ZO-2 fluorescent signal at TJs was reduced but was increased in the cytoplasm of ZO-1 knockout cells. (B) Effects of ZO-1 knockout on the localization of ZO-3 in MDCK II cells. ZO-3 fluorescent signal at TJs was slightly reduced but was increased in the cytoplasm of ZO-1 knockout cells. (C) Effects of ZO-1 knockout on the localization of ZO-2 in MDCK I cells. ZO-2 fluorescent signal was not altered in ZO-1 knockout MDCK I cells. (D) Effects of ZO-1 knockout on the localization of ZO-3 in MDCK I cells. ZO-3 fluorescent signal at TJs was markedly reduced in ZO-1 knockout MDCK I cells. Scale bar, 10 µm.

To generate TALEN DNA constructs for the knockout of ZO-1, we designed TALENs to target the left and right arms of the initiation codon of the canine ZO-1 gene (Fig. 1A) and constructed TALENs following a previously described method [27]. The TALEN constructs were transfected into MDCK II cells, a model cell line of renal proximal tubule cells. Immunofluorescence analysis of ZO-1 revealed the complete loss of ZO-1 staining in some regions, indicating the validity of the TALEN constructs for ZO-1 gene knockout in MDCK II cells. At the boundary of the control and ZO-1 knockout MDCK II cells, we observed characteristic convex curves of cell–cell junctions from knockout to control cells (Fig. 1B). In addition, ZO-2 and ZO-3 staining at TJs was reduced but was increased in the cytoplasm of ZO-1 knockout cells, especially for ZO-2 compared with ZO-3 (Fig. 2A and B).

We also performed ZO-1 knockout with TALEN constructs in MDCK I cells, a model cell line of renal distal tubule cells. The TALEN constructs also effectively induced ZO-1 knockout in MDCK I cells and similar morphological changes of cell–cell junctions at the boundary of control and ZO-1 knockout cells were observed (Fig. 1C). However, staining of ZO-2 at TJs was not altered in ZO-1 knockout MDCK I cells but ZO-3 staining at TJs was markedly reduced in ZO-1 knockout MDCK I cells compared with control cells (Fig. 2C and D). These results suggest ZO-1 plays a role in the regulation of cell–cell junction shape and localization of other ZO proteins.

### ZO-2 or ZO-3 knockout by TALEN-mediated gene targeting does not change cell–cell junction shape in MDCK II cells

**Figure 3:**
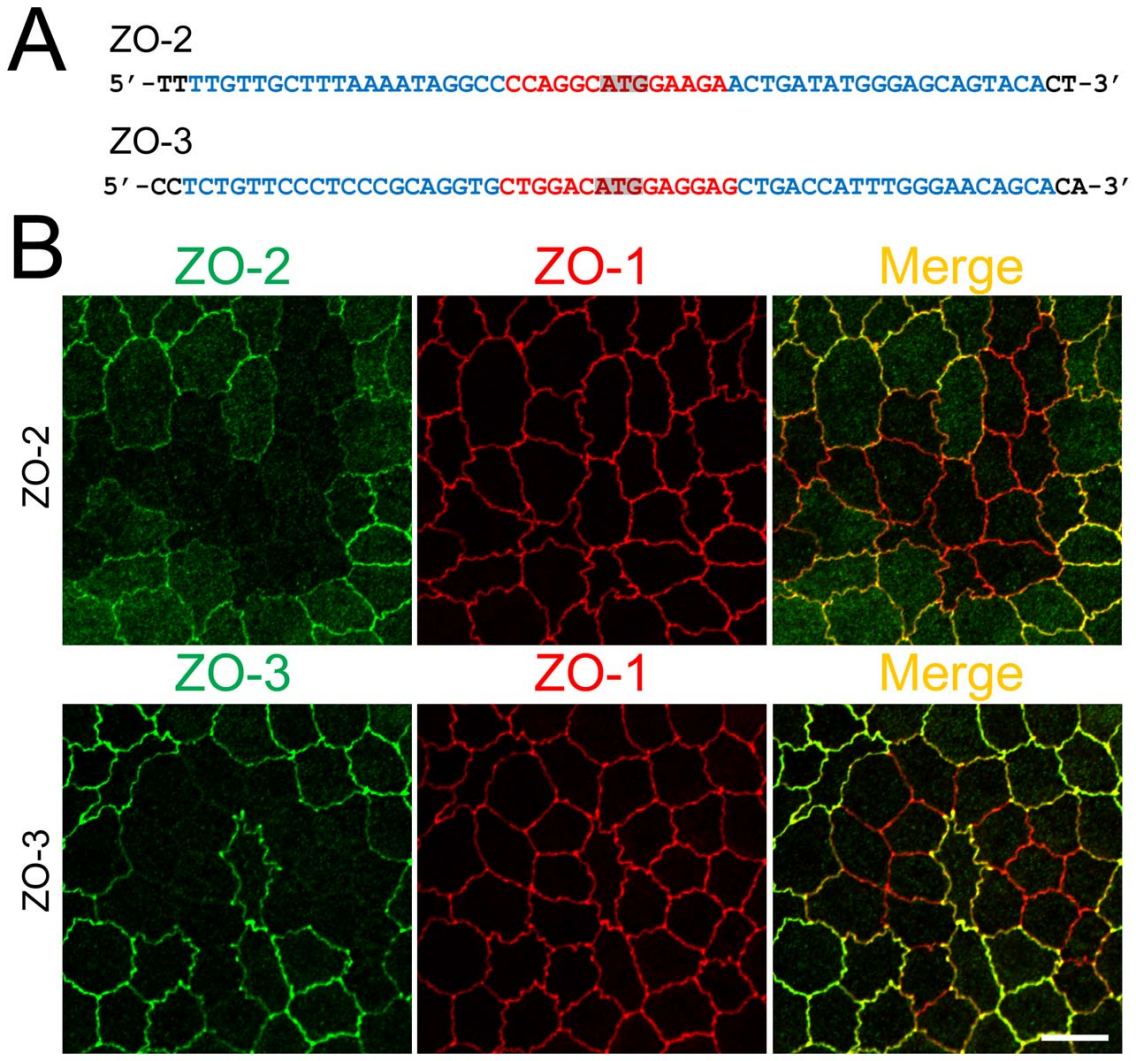
Knockout of ZO-2 or ZO-3 in MDCK II cells. (A) TALEN binding sites in ZO-2 and ZO-3 genes. The left and right arms of TALEN targeting sites are indicated in blue and the spacer region is indicated in red. The initiation codon within the spacer region is highlighted. (B) Immunofluorescence microscopic analysis of ZO proteins in MDCK II cells transfected with TALEN constructs for ZO-2 or ZO-3 gene knockout. The shape of cell–cell junctions was unchanged by ZO-2 knockout (upper panels) and ZO-3 knockout (lower panels). Scale bar, 10 µm.

To investigate the effects of the knockout of ZO proteins other than ZO-1, we constructed TALENs to knockout ZO-2 and ZO-3 similar to the construction of TALENs for ZO-1 (Fig. 3A). Immunofluorescence analysis of ZO-2 and ZO-3 in MDCK II cells revealed that TALENs effectively induced knockout of ZO-2 and ZO-3. Furthermore, the loss of ZO-2 staining was observed at the region of cell–cell contact but also at the cytoplasm in ZO-2 knockout cells, suggesting that ZO-2 staining in the cytoplasm was specific (Fig. 3B). In contrast to the changes observed by ZO-1 knockout, ZO-2 and ZO-3 knockout did not change cell–cell junction shape or the localization of ZO-1. These results suggest that ZO-1 affects cell–cell junction shape independently of ZO-2 and ZO-3.

### Establishment of ZO-1 knockout clones in MDCK II cells

To analyze the function of ZO-1 in MDCK II cells in detail, we attempted to establish ZO-1 knockout clones in MDCK II cells. We cloned each TALEN DNA construct for ZO-1 knockout into a mammalian expression vector with a neomycin resistance gene or puromycin resistance gene. Then we transfected the vectors into MDCK II cells and transiently administered (4 h) 500 µM G418 and 5 µM puromycin the next day after transfection to increase the selection efficiency for knockout clones. The remaining cell colonies containing ZO-1 negative cells were screened by immunofluorescence microscopy. After re-seeding these cells by limiting dilution, we succeeded in establishing three independent clones.

**Figure 4:**
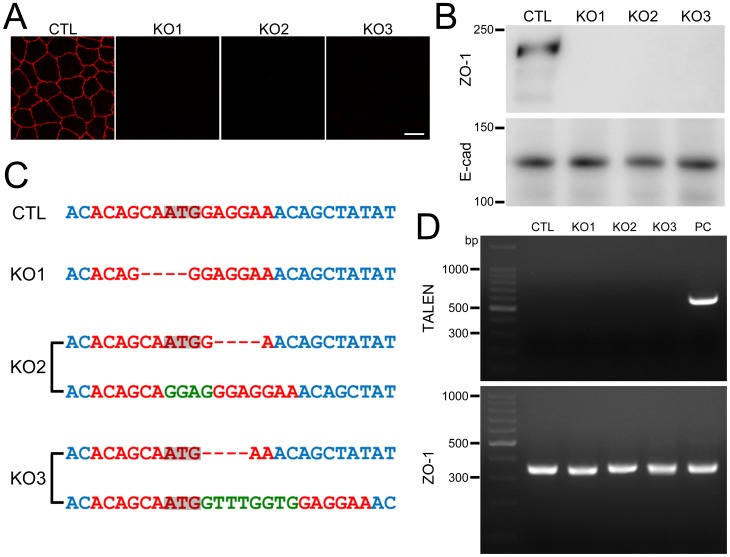
Establishment of ZO-1 knockout clones in MDCK II cells. (A) Immunofluorescence microscopic analysis of ZO-1 in control (CTL) MDCK II cells and ZO-1 knockout clones (KO 1–3). ZO-1 staining was completely lost in ZO-1 knockout clones. Scale bar, 10 µm. (B) Immunoblots of ZO-1 and E-cadherin (E-cad) in control MDCK II cells and ZO-1 knockout clones. Knockout clones showed no detectable bands of ZO-1. (C) DNA sequences of TALEN targeting sites in each allele of ZO-1 knockout clones. One type of mutation was present in the alleles of ZO-1 knockout clone 1 (KO 1) and two types of mutations in the alleles of clones 2 and 3 (KO 2 and 3). Dashes indicate loss of nucleotides and green letters indicate additional nucleotides. Loss of initiating codon or frameshift were confirmed in all alleles. (D) Genomic PCR analysis of control and ZO-1 knockout clones using primers for TALENs and ZO-1 DNAs. A clone stably expressing TALEN was used as a positive control (PC). None of the PCR products for TALENs were detected in ZO-1 knockout clones.

Immunofluorescence microscopic analysis showed a complete disappearance of ZO-1 staining and immunoblot analysis demonstrated complete elimination of ZO-1 expression in these clones (Fig. 4A and B). To confirm mutations of the TALEN targeting site in the ZO-1 gene, PCR products of this site from the clones were directly subjected to DNA sequencing analysis. The chromatograms of sequences showed a single peak array in clone 1 and mixed peak arrays in clones 2 and 3 (Fig. S2). We therefore cloned PCR products of clones 2 and 3 into a plasmid vector for sequence analysis. We found two patterns in the chromatograms of sequences for clones 2 and 3, and a comparison of their peak arrays revealed their mixed peak arrays were composed of these two chromatograms, respectively (Fig. S2). These results indicated that TALENs targeting ZO-1 induced the same ZO-1 allele mutations for clone 1 and two types of mutations in the alleles of ZO-1 for clones 2 and 3. Analysis of the sequencing results for each allele revealed loss of the initiating codon, or a frameshift in all alleles (Fig. 4C). These results indicated successful ZO-1 gene knockout in these clones.

We also confirmed whether the TALEN constructs transfected into the clones were integrated into the chromosome. We performed genomic PCR using primers for the TALEN C-terminal region. Clones stably expressing TALEN were established by selection with the persistent administration of G418, and one of these clones was used as a positive control. None of the PCR products of these clones showed detectable 558 bp bands of TALEN (Fig. 4D), suggesting that the TALEN constructs were not integrated into the chromosome in these clones.

### ZO-1 knockout induces striking changes in myosin organization at cell–cell contacts

**Figure 5:**
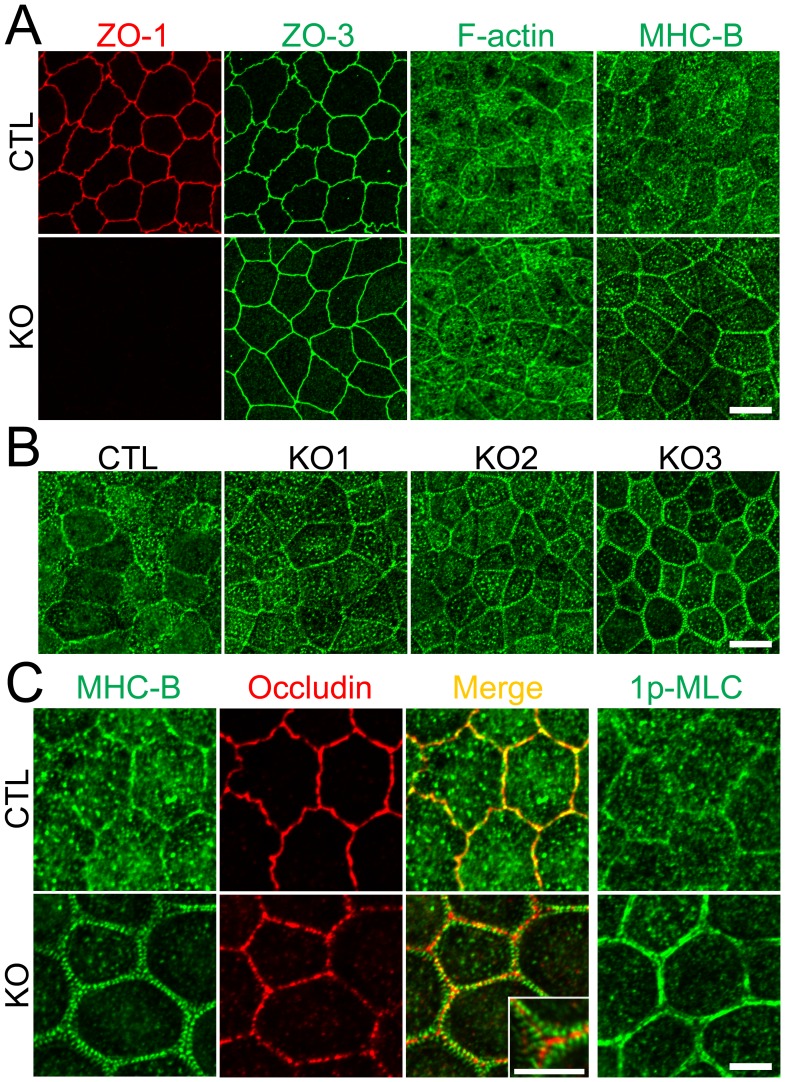
Effects of ZO-1 knockout on the shape of cell–cell junctions and cytoskeleton. (A) Immunofluorescence microscopic analysis of ZO-1, ZO-3, F-actin and myosin heavy chain II-B (MHC-B) in control MDCK II cells and ZO-1 knockout clone. ZO-1 knockout induced striking changes in MHC-B organization. Scale bar, 10 µm. (B) Immunofluorescence microscopic analysis of MHC-B in control MDCK II cells and ZO-1 knockout clones. Similar punctate staining of MHC-B at cell–cell contacts was observed in all three ZO-1 knockout clones. Scale bar, 10 µm. (C) Immunofluorescence microscopic analysis of MHC-B, occludin and 1-phosphomyosin light chain (1p-MLC) at high magnification. Coimmunolocalization of MHC-B and occludin revealed punctate staining of MHC-B was arranged in two rows along the cell–cell junctions across the staining of occludin. 1p-MLC was concentrated at cell–cell contacts in ZO-1 knockout cells. Scale bar, 5 µm.

To compare the effects of ZO-1 knockout on cell–cell junction shape and cytoskeleton with findings in previous ZO-1 knockdown studies [13]–[15], we examined the distribution of ZO-3, F-actin and myosin heavy chain B (MHC-B) by immunofluorescence microscopy. ZO-1 knockout altered cell–cell junction shape from zigzag to straight and slightly increased actin staining at the perijunctional region (Fig. 5A) in agreement with a previous study reported by Van Itallie et al. [13]. In addition, more striking changes of MHC-B were observed in ZO-1 knockout cells (Fig. 5A). We found obvious punctate staining of MHC-B at cell–cell contacts and these changes were apparent 4 days after seeding on filter inserts. Similar punctate staining of MHC-B at cell–cell contacts was observed in all three ZO-1 knockout clones, although there was a variation in the degree of punctate patterns (Fig. 5B). Close observation at high magnification revealed that punctate staining of MHC-B was arranged in two rows along the cell–cell junctions across the staining of occludin, a transmembrane protein of TJs, in ZO-1 knockout cells (Fig. 5C).Furthermore, 1-phosphomyosin light chain (1p-MLC), an active form of myosin light chain, was also concentrated at the cell–cell contacts in ZO-1 knockout cells.

**Figure 6:**
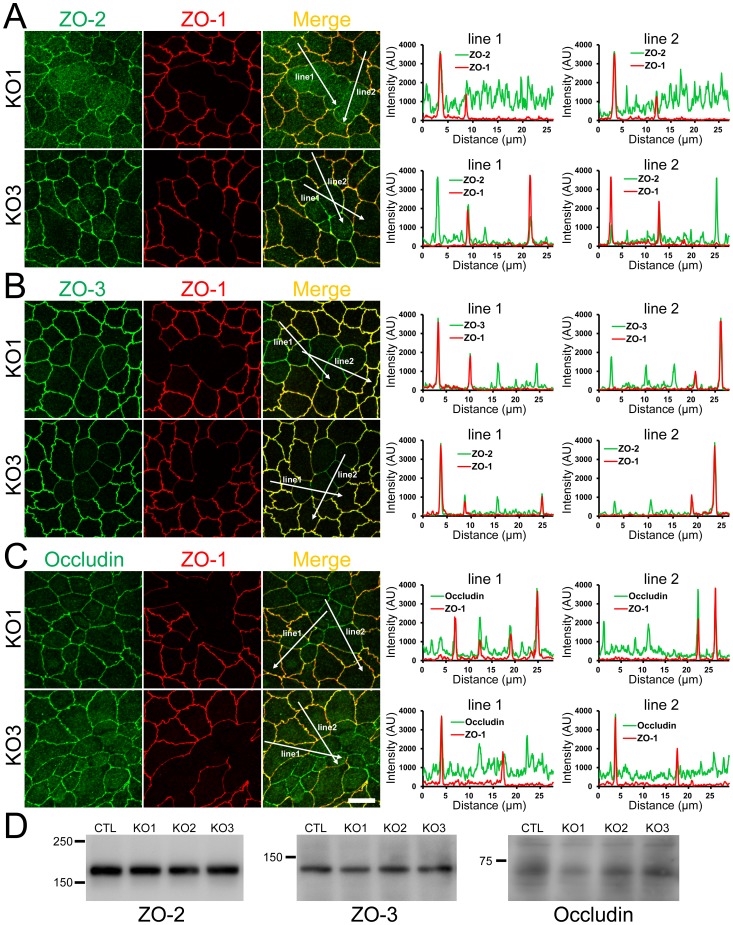
Effects of ZO-1 knockout on the localization of ZO-2, ZO-3 and occludin in the stable ZO-1 knockout clones. (A) Immunofluorescence microscopic analysis and line scanning of ZO-1 and ZO-2 in ZO-1 knockout clones 1 (KO 1) and 3 (KO 3) co-cultured with control cells. ZO-2 fluorescent signal at TJs was reduced in KO 1 but was slightly increased in KO 3. (B) Immunofluorescence microscopic analysis and line scanning of ZO-1 and ZO-3. ZO-3 signal at TJs was reduced but was increased in the cytoplasm in both of ZO-1 knockout clones compared to co-cultured control cells. (C) Immunofluorescence microscopic analysis and line scanning of ZO-1 and occludin. Occludin signal at TJs was reduced in both of ZO-1 knockout clones compared to co-cultured control cells. Scale bar, 10 µm. (D) Immunoblots of ZO-2, ZO-3 and occludin in control MDCK II cells and ZO-1 knockout clones. Similar expression levels of ZO-2, ZO-3 and occludin were observed in control cells and knockout clones.

**Figure 7:**
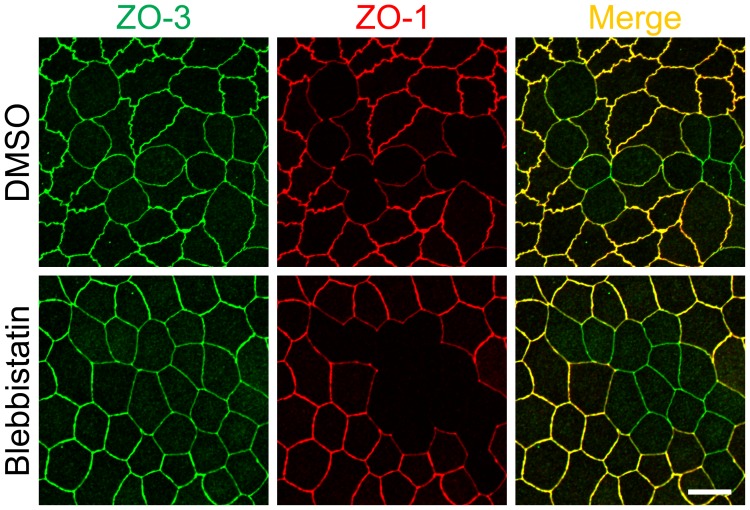
Effects of blebbistatin on the shape of cell-cell junctions in ZO-1 knockout cells. Effects of blebbistatin on the shape of cell-cell junctions in the co-culture of control and ZO-1 knockout cells. Cells were treated with 50 µM blebbistatin for 2 h and analyzed by immunofluorescence microscopy. Blebbistatin treatment attenuated the curves of cell-cell junctions at the boundary of the control and ZO-1 knockout cells, and the shape of cell-cell junctions in the control and ZO-1 knockout cells were almost indistinguishable in blebbistatin treated cells. Scale bar, 10 µm.

We also examined the distribution of ZO-2, ZO-3 and occludin in ZO-1 knockout clones in the co-culture with control MDCK II cells. ZO-2 staining at TJs was reduced in knockout clone 1 consistent with the results in Figure 1 and 2 but was slightly increased in knockout clone 3 (Fig. 6A), indicating the variation in the effects of ZO-1 knockout on ZO-2 localization between knockout clones. In contrast, ZO-3 and occludin staining at TJs were reduced in ZO-1 knokcout cells of both clones (Fig. 6B and C). Immunoblot analysis showed similar expression levels of ZO-2, ZO-3 and occludin in control and knockout cells (Fig. 6D). These results indicate that ZO-1 knockout affects the localization of ZO-2, ZO-3 and occludin but does not change the expression levels of these proteins. We also observed the characteristic curves of cell-cell junctions at the boundary of control and ZO-1 knockout cells similar to the results in Figure 1 and 2. Since ZO-1 knockout induced striking changes in myosin organization (Fig. 5), we examined the effects of myosin II ATPase inhibitor blebbistatin on the shape of cell-cell junctions. Blebbistatin treatment attenuated the curves of cell-cell junctions at the boundary of the control and ZO-1 knockout cells, and the shape of cell-cell junctions in the control and ZO-1 knockout cells were almost indistinguishable in blebbistatin treated cells (Fig. 7). These results suggest that the differences of the tension at cell-cell contacts generated by myosin activity between control and ZO-1 knockout cells may cause the characteristic convex curves of cell-cell junctions at the boundary of control and ZO-1 knockout cells.

### Effects of ZO-1 expression levels on cell–cell contact shape and cytoskeleton

**Figure 8:**
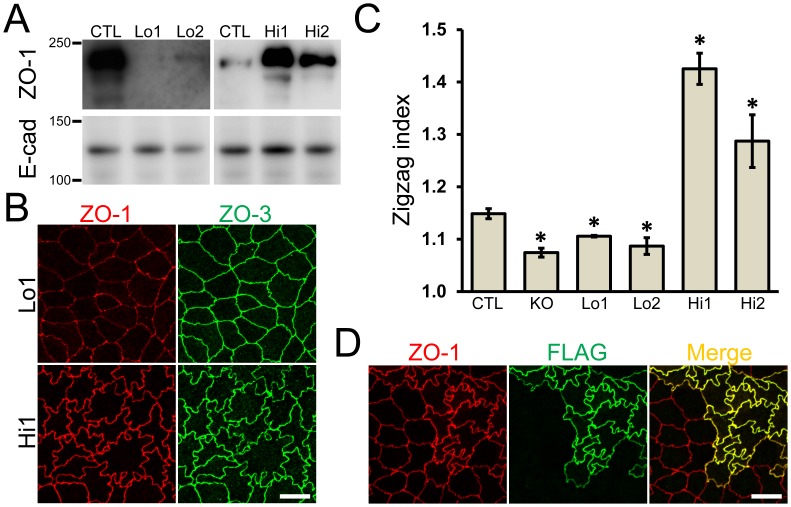
Effects of the amount of ZO-1 expression on the shape of cell–cell junctions. (A) Immunoblots of ZO-1 and E-cadherin in control MDCK II cells and ZO-1 knockout clones expressing exogenous ZO-1. FLAG-tagged ZO-1 was transfected into ZO-1 knockout cells, and clones expressing a nominal amount of ZO-1 (Lo 1 and 2) and an excessive amount of ZO-1 (Hi 1 and 2) were established. The bands of ZO-1 from Lo 1 and 2 clones were detected at very low levels in the enhanced image of immunoblots incubated with anti-ZO-1 antibody. (B) Immunofluorescence microscopic analysis of ZO-1 and ZO-3 in clones Lo 1 and Hi 1. Intensive zigzag shape of cell–cell junctions was observed in Hi 1 clone. Scale bar, 10 µm. (C) Effect of ZO-1 expression levels on the shape of cell–cell junctions. The degree of zigzag of cell–cell junctions was quantified in each clone and presented as zigzag index. The zigzag index was significantly lower in ZO-1 knockout clones and Lo 1 and 2 clones, and was significantly higher in Hi 1 and 2 clones compared with control cells. Data are shown as means ± S.E. N = 3–4 for each clone. *, *p*<0.05 compared with control. (D) Immunofluorescence microscopic analysis of ZO-1 and FLAG-tagged ZO-1. Control MDCK II cells and Hi 1 cells were co-cultured on filter inserts. Hi 1 cells expressing FLAG-tagged ZO-1 have an intensive zigzag cell–cell contact shape. Scale bar, 10 µm.

Next, we performed rescue experiments using mouse ZO-1 cDNA tagged with FLAG at the N-terminus. To examine the effect of ZO-1 expression levels, we transfected mouse ZO-1 cDNA into ZO-1 knockout clones and established subclones expressing trace amounts of ZO-1 (Lo 1 and 2) and excessive levels of ZO-1 (Hi 1 and 2). By immunoblot analysis, ZO-1 bands in Lo 1 and 2 clones were barely detectable by enhanced imaging (Fig. 8A) and ZO-1 staining was only observed at cell–cell junctions by enhanced images by immunofluorescence microscopy (Fig. 8B). The zigzag cell–cell junction shape was slightly recovered in Lo 1 and 2 clones, but the degree of zigzag (zigzag index) was still significantly lower than in control cells (Fig. 8C). In contrast, the Hi 1 and Hi 2 clones with excessive ZO-1 expression had an intensive zigzag cell–cell junction shape (Fig. 8B and Fig. S3). A co-culture experiment showed definitive changes of the cell–cell junction shape in cells expressing excessive levels of ZO-1 tagged with FLAG (Fig. 8D), and the zigzag index indicated they were significantly higher in these clones than in the control cells (Fig. 8C). These results indicate that ZO-1 expression levels affect the degree of cell–cell junction zigzag in MDCK II cells.

**Figure 9:**
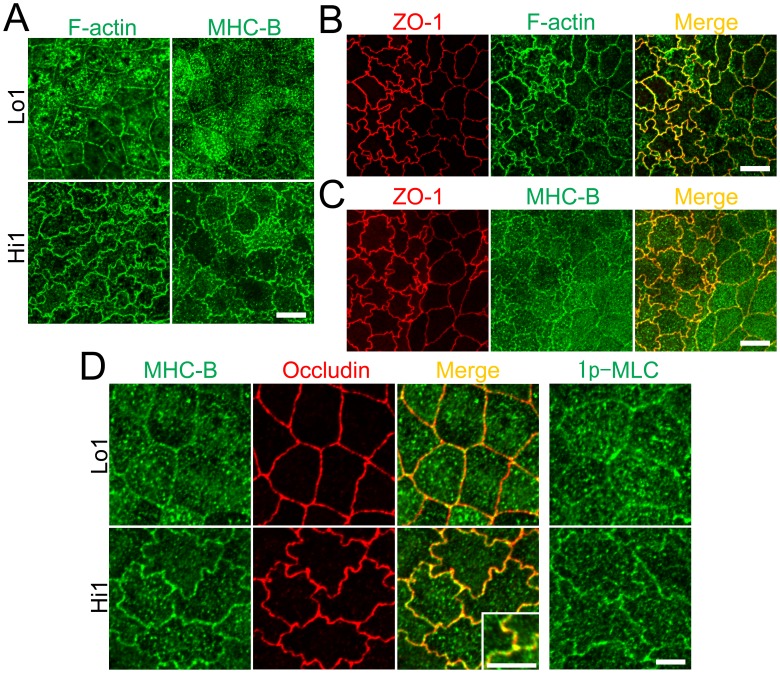
Effect of ZO-1 expression levels on the cytoskeleton. (A) Immunofluorescence microscopic analysis of F-actin and MHC-B in Lo 1 and Hi 1 clones. The organization of MHC-B is recovered in Lo 1 clone. The assembly of F-actin and MHC-B at cell–cell contacts was observed in the Hi 1 clone. Scale bar, 10 µm. (B and C) Immunofluorescence microscopic analysis of ZO-1, F-actin and MHC-B in the co-culture of control MDCK II cells and Hi 1 cells. Assemblies of F-actin and MHC-B at cell–cell contacts were observed in cells expressing an excess amount of ZO-1. Scale bar, 10 µm. (D) Immunofluorescence microscopic analysis of MHC-B, occludin and 1-pMLC at high magnification in Lo 1 and Hi 1 clones. Coimmunolocalization of MHC-B and occludin revealed an overlap of MHC-B and occludin staining. 1p-MLC was assembled at cell–cell contacts in Hi 1 cells. Scale bar, 5 µm.

We also examined the distribution of F-actin and MHC-B in these clones. Although the degree of zigzag was significantly lower in Lo 1 and 2 clones than control cells, the nominal expression of ZO-1 could recover the organization of MHC-B (Fig. 9 and Fig. S3). The localization of occludin was also recovered by nominal expression of ZO-1 (Fig. 9D). We unexpectedly observed obvious zigzag staining of F-actin and MHC-B in Hi 1 and 2 clones (Fig. 9, and Fig. S3). Co-culture experiments revealed increased assembly of F-actin and MHC-B at cell–cell contacts in Hi 1 cells compared with control cells (Fig. 9B and C). Close observation of Hi 1 cells at high magnification showed the overlap of MHC-B and occludin staining, and the assembly of 1p-MLC at cell–cell contacts (Fig. 9D). These results indicate that the nominal expression of ZO-1 is sufficient to rescue myosin organization but insufficient to rescue cell–cell contact shape caused by ZO-1 knockout. Furthermore, the excessive expression of ZO-1 induced an intensive zigzag cell–cell junction shape accompanied by the assembly of F-actin and myosin at cell–cell contacts.

### Time-lapse imaging of fluorescent protein Venus-ZO-1 in MDCK II cells

**Figure 10:**
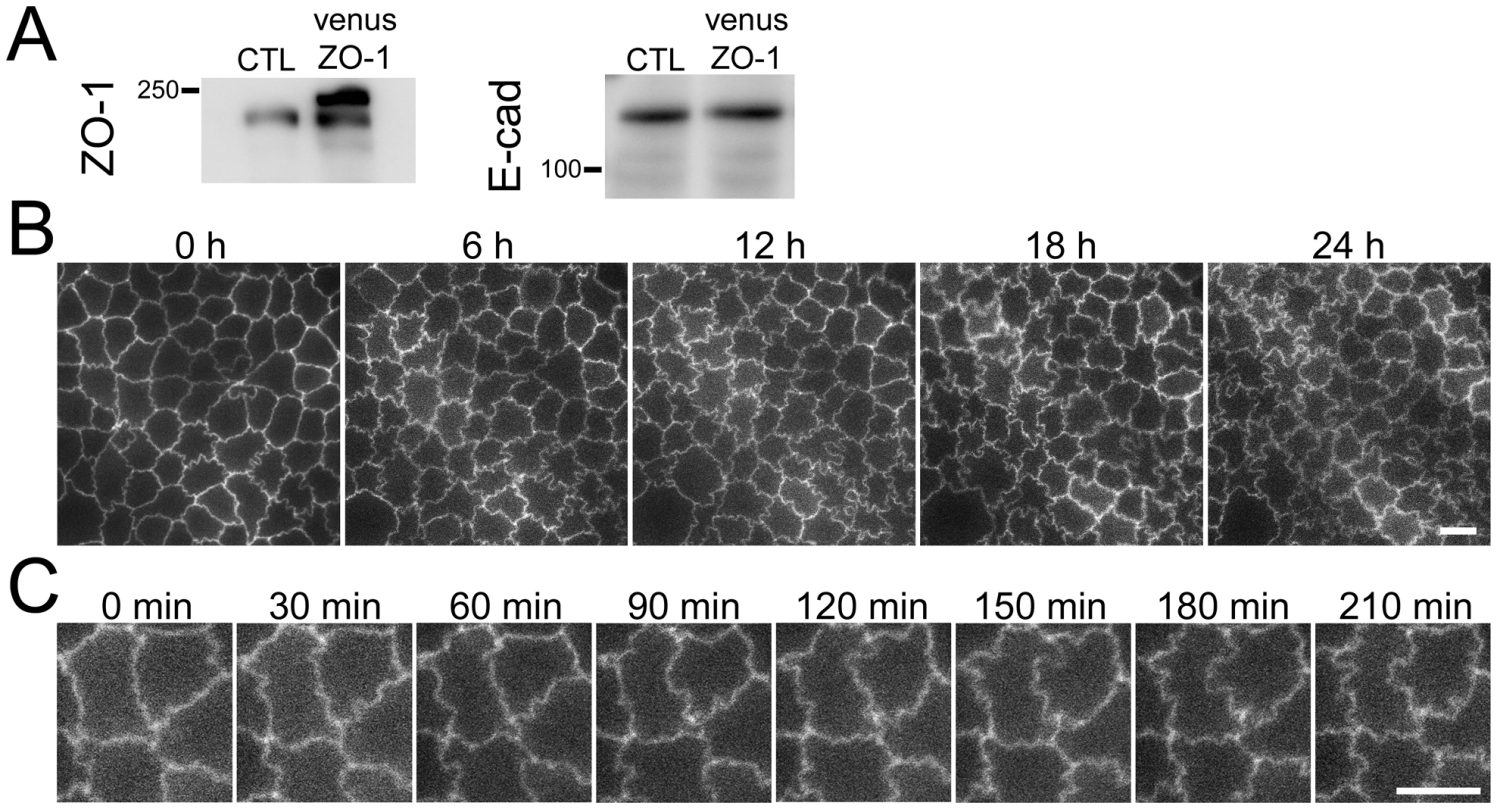
Time-lapse imaging of Venus-ZO-1 expressing MDCK II cells. (A) ZO-1 and E-cadherin in control and Venus-ZO-1 expressing MDCK II cells. ZO-1 tagged with fluorescent protein Venus was transfected into control MDCK II cells, and stably expressing clones were established. Expression of excessive levels of Venus-ZO-1 was detected as a band with higher molecular weight in addition to endogenous ZO-1 in this clone. (B and C) Time lapse-imaging of Venus-ZO-1 expressing MDCK cells. Cells were cultured on the reverse side of filter inserts for 3 d, and time-lapse images were collected every 30 min for 24 h. The shape of cell–cell junctions showed gradually increasing degree of zigzag with continual changes in shape. Scale bar, 10 µm.

To observe the formation of intensive zigzag cell–cell junction shapes, we transfected mouse ZO-1 cDNA tagged with a fluorescent protein Venus into wild MDCK II cells, and established a stable expressing clone (Fig. 10A). We also confirmed the intensive zigzag cell–cell junction shape in these cells, indicating that this phenotype is independent of the type of tags used. Three days after the seeding of Venus-ZO-1 expressing MDCK II cells on filter inserts, we conducted time-lapse imaging of fluorescent Venus signal for 24 h at 30 min intervals. The degree of zigzag shape of cell–cell junctions gradually increased with continual changes in their shape over 30 min sequences (Fig. 10B and C; Movie S1), indicating the dynamic property of cell–cell junction shapes in these cells.

### Effects of ZO-1 knockout on TJ barrier function

**Figure 11:**
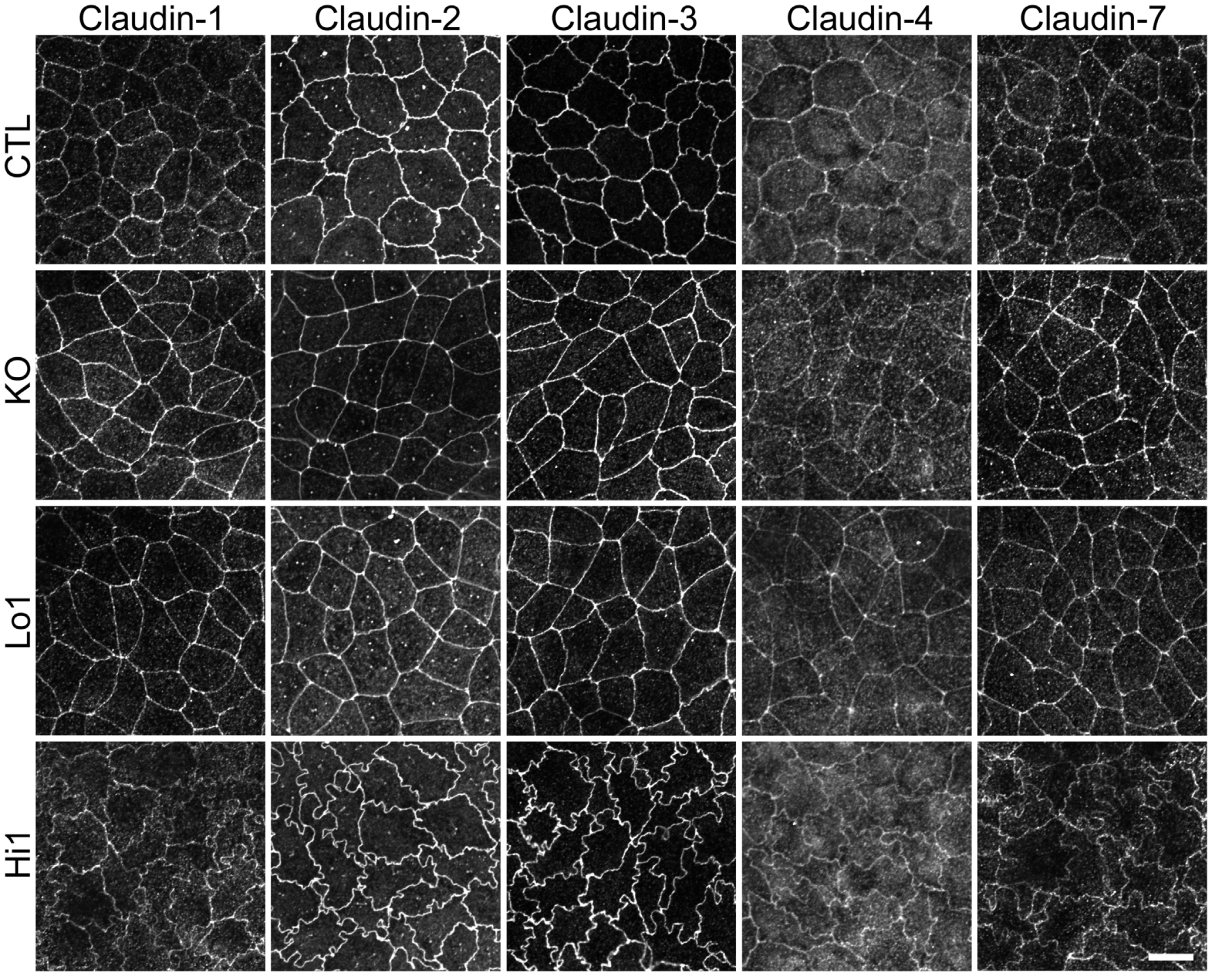
The localization of claudins in ZO-1 knockout, Lo 1 and Hi 1 clones. Immunofluorescence microscopic analysis of claudin-1, -2, -3, -4 and -7 in control cells and ZO-1 knockout, Lo 1 and Hi 1 clones. Claudin-2 staining at TJs was reduced but claudin-1 and claudin-7 staining at TJs were increased in ZO-1 knockout cells, and a nominal expression of ZO-1 (Lo 1) restored these changes. Claudin-3, -4 and -7 staining were similar in control, ZO-1 knockout, Lo 1 and Hi 1 cells. Scale bar, 10 µm.

**Figure 12:**
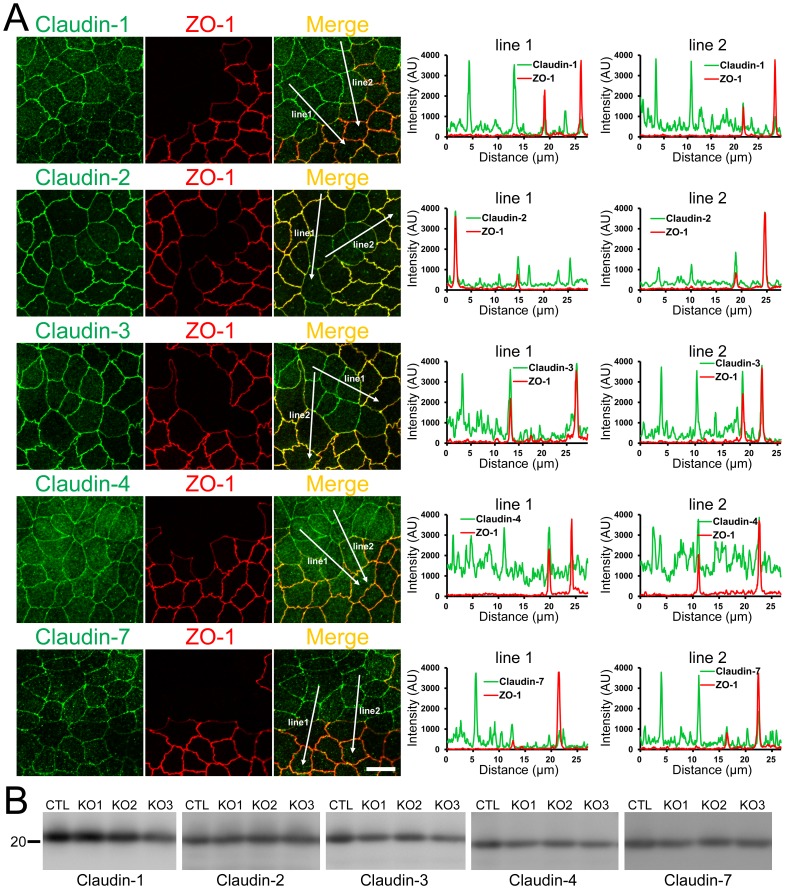
Effects of ZO-1 knockout on the localization and expression levels of claudins. (A) Effects of ZO-1 knockout on the localization of claudins. Control and ZO-1 knockout cells of clone 1 were co-cultured on filter inserts. Signal intensity of claudins on lines shown in confocal microscopic images (*arrows*) were analyzed. Claudin-2 fluorescent signal at TJs was increased but claudin-1 and -7 signals at TJs were reduced in ZO-1 knockout cells. Scale bar, 10 µm. (B) Immunoblots of claudins in control MDCK II cells and ZO-1 knockout clones. Similar expression levels of claudins were observed in control and knockout cells.

**Figure 13:**
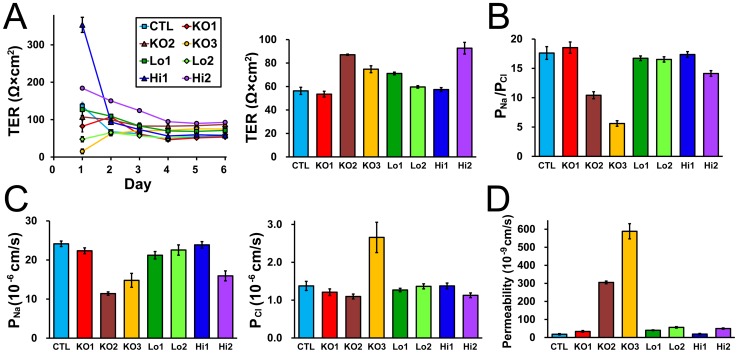
Effects of ZO-1 knockout on the barrier properties of TJs. (A) Time course of TER and TER values 6 days after the seeding in control cells and ZO-1 knockout and rescue clones. ZO-1 overexpressing clones (Hi 1 and 2) showed higher TER values on the next day of the seeding on filter inserts than other clones, but TER showed steady values after 4 days of seeding in all clones. TER values 6 days after the seeding was not changed in ZO-1 knockout clone 1 (KO 1) but increased in clones 2 (KO 2) and 3 (KO 3) compared with control cells. (B) Charge selectivity (the ratio of *P*_Na_ and *P*_Cl_: *P*_Na_/*P*_Cl_) in control cells and ZO-1 knockout and rescue clones. *P*_Na_/*P*_Cl_ was similar in ZO-1 knockout clone 1 whereas were decreased clones 2 and 3. (C) *P*_Na_ and *P*_Cl_ in control cells and ZO-1 knockout and rescue clones. *P*_Na_ was decreased in ZO-1 knockout clones 2 and 3 and *P*_Cl_ was increased in clone 3. (D) The flux of 4 kDa FITC-dextran in control cells and ZO-1 knockout and rescue clones. ZO-1 knockout clone 1 showed small, but significant, increase in the flux of 4 kDa dextran compared with control cells. In contrast, the flux of 4 kDa dextran in clone 2 and 3 were more than 10 times higher than control cells. Data are shown as means ± S.D. N = 4 for each experiment.

We also examined the effects of ZO-1 knockout on TJ barrier function. The barrier property of TJs is known to be regulated by claudins [33], therefore we first observed the localization of claudins in MDCK II cells. Claudin-1, -2, -3, -4 and -7 were detected by our antibodies consistent with the previous study [34]. Claudin-2 staining at TJs was reduced but claudin-1 and claudin-7 staining at TJs were increased in ZO-1 knockout cells, and a nominal expression of ZO-1 restored these changes (Fig. 11). These effects of ZO-1 knockout on claudin localization were more clearly confirmed in co-culture experiments of control and ZO-1 knockout cells of clone 1 (Fig. 12A) and clone 3 (Fig. S4). Immunoblot analysis showed similar expression levels of claudins in control and ZO-1 knockout cells (Fig. 12B). These results indicate that ZO-1 knockout affects the localization of claudins but does not change the expression levels of these proteins. Then we measured transepithelial electrical resistance (TER) to assess the ionic permeability of TJs. ZO-1 overexpressing clones (Hi 1 and 2) showed higher TER values on the next day of the seeding on filter inserts than other clones, but TER showed steady values after 4 days of the seeding in all clones (Fig. 13A). TER values 6 days after the seeding were variable between clones; TER value was not changed in ZO-1 knockout clone 1 but increased in clones 2 and 3 compared with control cells. We also measured Na^+^ permeability (*P*_Na_) and Cl^−^ permeability (*P*_Cl_) of TJs. Control MDCK II cells showed very high cation selectivity (the ratio of *P*_Na_ and *P*_Cl_: *P*_Na_/*P*_Cl_) consistent with the previous studies [34], [35], and *P*_Na_/*P*_Cl_ was similar in control cells and ZO-1 knockout clone 1 whereas were decreased in clones 2 and 3 (Fig. 13B). *P*_Na_ was decreased in ZO-1 knockout clones 2 and 3 and *P*_Cl_ was increased in clone 3 (Fig. 13C). The flux of 4 kDa FITC-dextran, the non-charged large molecule, also showed large variance between clones. ZO-1 knockout clone 1 showed small, but significant, increase in the flux of 4 kDa dextran compared with control cells. In contrast, the flux of 4 kDa dextran in clone 2 and 3 were more than 10 times higher than control cells (Fig. 13D). We could not conclude the consistent tendency of the effects of ZO-1 knockout on TJ barrier function.

## Discussion

In this study, we succeeded in establishing ZO-1 knockout clones in MDCK II cells using the TALEN technique. To improve the efficiency of the selection of knockout clones, we cloned a pair of TALEN constructs into neomycin and puromycin resistant expression vectors, respectively, for the selection of cells into which both TALEN constructs were transfected. We applied transient but not persistent administration of G418 and puromycin after the transfection of these vectors into MDCK II cells to avoid selection of clones in which TALEN constructs were integrated into the chromosome. This method allowed us to establish three independent ZO-1 knockout clones. Genomic PCR analysis indicated that TALEN DNAs were not integrated into the chromosome in these clones. Since the persistent expression of TALENs has potential adverse effects such as increased frequency of off-target cleavage [36], the establishment of knockout clones in which TALENs are not integrated into the chromosome is ideal. This is likely to be more important for double and triple knockout genes by TALENs, because persistent TALEN expression in cells can form *Fok*I dimers at off-target sites with TALENs transfected for the knockout of another gene. We need to check whether we can establish knockout clones of genes other than ZO-1 by the method used in this study and assess the rate of the TALEN integration into the chromosome in future studies. In addition, we found a variation in the degree of myosin reorganization, ZO-2 localization and TJ barrier function between the three ZO-1 knockout clones, indicating the need for analysis in several clones even when using gene knockout methods.

Several previous studies reported the effects of ZO-1 knockdown by RNAi in MDCK II cells [13]–[15]. Van Itallie et al., reported that ZO-1 knockdown changed the shape of cell–cell junctions from tortuous to linear with reorganization of the perijunctional cytoskeleton, while two other studies did not note these changes. In the current study, we found that ZO-1 knockout changed the shape of cell–cell junctions and F-actin in agreement with the study reported by Van Itallie et al. [13]. Moreover, ZO-1 knockout induced more striking changes in myosin organization and also affected the localization of occludin, ZO-2, ZO-3 and claudins at TJs in MDCK II cells. Since a trace amount of ZO-1 expression recovered changes of myosin organization and occludin and claudins localization, differences between studies might be explained by the effects of remaining low level ZO-1 expression in the previous study. Interestingly, the striking changes of myosin organization induced by ZO-1 knockout closely resembled those induced by the double-knockdown of ZO-1 and ZO-2 in MDCK cells [37]. Disruption of the localization of occludin and claudin-2 at TJs was also observed in ZO-1 and ZO-2 double-knockdown cells similar to that in ZO-1 knockout cells. Because ZO-2 knockdown alone did not affect the cytoskeleton or localization of TJ proteins [13], ZO-2 is unlikely to regulate these cell functions independently. It is premature to discuss the association between observations in ZO-1 knockout and ZO-1 and ZO-2 double-knockdown cells at present. Further analysis of the effects of ZO-2 knockout and ZO-1 and ZO-2 double-knockout on MDCK cell functions is required. On the other hand, we did not observe changes in the apical surface of cells in ZO-1 knockout cells (unpublished data), which was observed in a previous study [37], indicating that ZO-1 and ZO-2 are functionally redundant for this function.

In contrast to the linear shape of cell–cell junctions in ZO-1 knockout cells, the excessive expression of ZO-1 induced an intensive zigzag shape of cell–cell junctions, suggesting that the amount of ZO-1 expression is one of the factors that determine the degree of zigzag of cell–cell junctions in MDCK II cells. Because the degree of zigzag of cell–cell junctions correlates with an area of paracellular pathway and consequently increases the overall permeability of the epithelia [38], ZO-1 expression levels are thought to affect the epithelial permeability by changing the cell–cell junction shape in MDCK II cells. However, we could not found consistent tendency of the relationship between ZO-1 expression levels and TJ barrier function including TER and flux measurements because of the large variance between clones. Further analysis by the rescue experiments in several ZO-1 knockout clones with inducible expression of ZO-1 are required in future studies. Despite a large difference in the cell shape of cell–cell junctions, the assembly of F-actin and myosin at cell–cell contacts were observed in both ZO-1 knockout and ZO-1 overexpressing cells, although the localization of myosin was slightly different between these cells (Figs. 5 and 9). The assembly of the cytoskeleton at cell–cell contacts was reported to be regulated by several proteins including the apical junctional complex-associated scaffold protein Shroom3, the FERM-domain protein Lulu and cdc42 GEF Tuba, which increase tension at cell–cell contacts resulting in a linear shape of cell–cell junctions [39]–[41]. However, our observations in ZO-1 overexpressing cells indicate that the extent of the accumulation of F-actin and myosin at cell–cell contacts does not necessarily correlate with the linear shape of cell–cell junctions. We do not know whether the cytoskeleton assembled at cell–cell contacts is associated with the formation of the zigzag shape of cell–cell junctions, and if so, how the cytoskeleton regulates the zigzag shape in ZO-1 overexpressing cells. Because ZO-1 binds many regulatory components of the cytoskeleton [8], [12], investigation of the effects of ZO-1 expression levels on these components and a more detailed morphological analysis are required.

The role of ZO proteins in the formation of TJs remains controversial. ZO-depleted EpH4 cells by ZO-1 knockout and ZO-2 knockdown resulted in the complete loss of TJs [17], while ZO-depleted MDCK cells by double-knockdown of ZO-1 and ZO-2 had functional TJ strands although the property of the TJ barrier was altered [37]. Reasons for differences between these studies are likely explained by the ZO-1 depletion method and/or different cell types used. To clearly demonstrate the role of ZO proteins in the formation of TJs, it is needed to examine the formation of TJs in ZO-1 and ZO-2 double-knockout MDCK cells, and we are now conducting these experiments.

In summary, we established ZO-1 knockout clones in MDCK II cells and revealed new insights into the role of ZO-1 in regulation of the cytoskeleton and cell shape at cell–cell contacts. Our study also implicates the effectiveness of analysis by gene knockout in cultured cells.

## Supporting Information

Figure S1 **Quantification of the degree of zigzag of cell–cell junctions.** Stacked confocal images of ZO-3 were processed in Image J 1.43u and all sides contained in the area were traced with freehand lines (L_TJ_) or straight lines (L_St_) and the zigzag index was determined.
(TIF)

Figure S2 **Chromatograms of sequences around TALEN targeting site in control and ZO-1 knockout clones.** PCR products of TALEN targeting sites from control and ZO-1 knockout clones were directly subjected to DNA sequencing analysis (control, CTL; knockout, KO 1–3). Chromatograms of sequences for KO 2 and 3 clones showed mixed peak arrays, thus PCR products from KO 2 and 3 clones were cloned into a plasmid vector and subjected to sequence analysis (allele 1 and 2 for each clone).
(TIF)

Figure S3 **Effect of ZO-1 expression levels on the shape of cell–cell junctions and cytoskeleton in Lo 2 and Hi 2 clones.** Immunofluorescence microscopic analysis of ZO-1, ZO-3, F-actin and MHC-B in Lo 2 and Hi 2 clones.
(TIF)

Figure S4 **Effects of ZO-1 knockout on the localization and expression levels of claudins in ZO-1 knockout clone 3.** (A) Effects of ZO-1 knockout on the localization of claudins. Control and ZO-1 knockout cells of clone 3 were co-cultured on filter inserts. Signal intensity of claudins on lines shown in confocal microscopic images (*arrows*) were analyzed. Claudin-2 fluorescent signal at TJs was increased but claudin-1 and -7 signals at TJs were reduced in ZO-1 knockout cells. (B) Immunoblots of claudins in control MDCK II cells and ZO-1 knockout clones. Similar expression levels of claudins were observed in control and knockout cells.
(TIF)

Movie S1 **Time-lapse imaging of Venus-ZO-1 expressing MDCK II cells.** MDCK II cells expressing Venus-ZO-1 were cultured on the reverse side of filter inserts for 3 d, and time-lapse images were collected every 30 min for 24 h. The shape of cell–cell junctions show gradually increasing degree of zigzag with continual changes in shape.
(AVI)
