# Supplementary figures and images for: ZO-1 Knockout by TALEN-Mediated Gene Targeting in MDCK Cells: Involvement of ZO-1 in the Regulation of Cytoskeleton and Cell Shape
Source: PLoS One. 2014 Aug 26;9(8):e104994. doi: 10.1371/journal.pone.0104994 (PMC4144852; doi:10.1371/journal.pone.0104994)

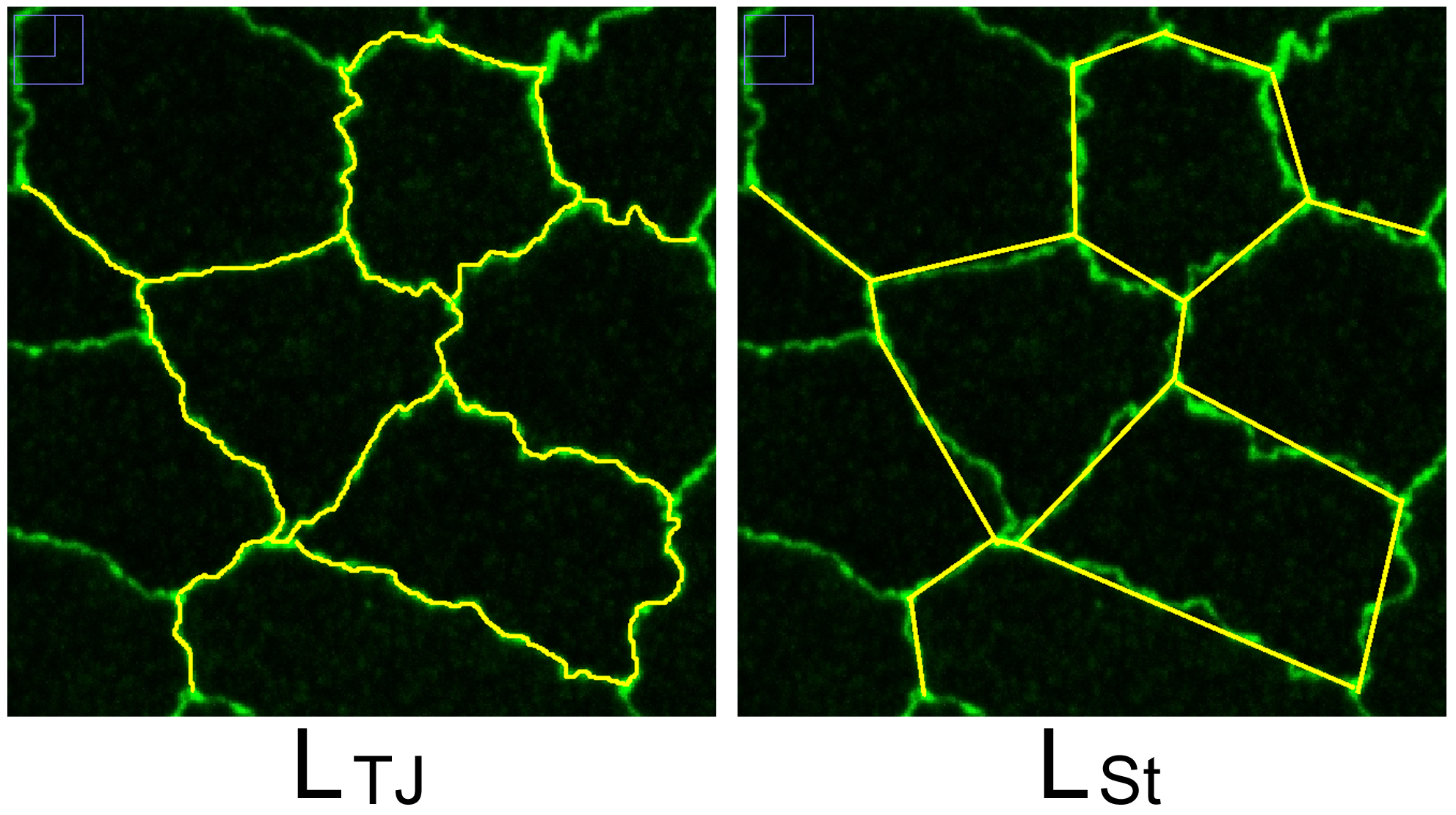

Supplement: Figure S1 — Quantification of the degree of zigzag of cell–cell junctions. Stacked confocal images of ZO-3 were processed in Image J 1.43u and all sides contained in the area were traced with freehand lines (LTJ) or straight lines (LSt) and the zigzag index was determined. (TIF) [file pone.0104994.s001.tif]

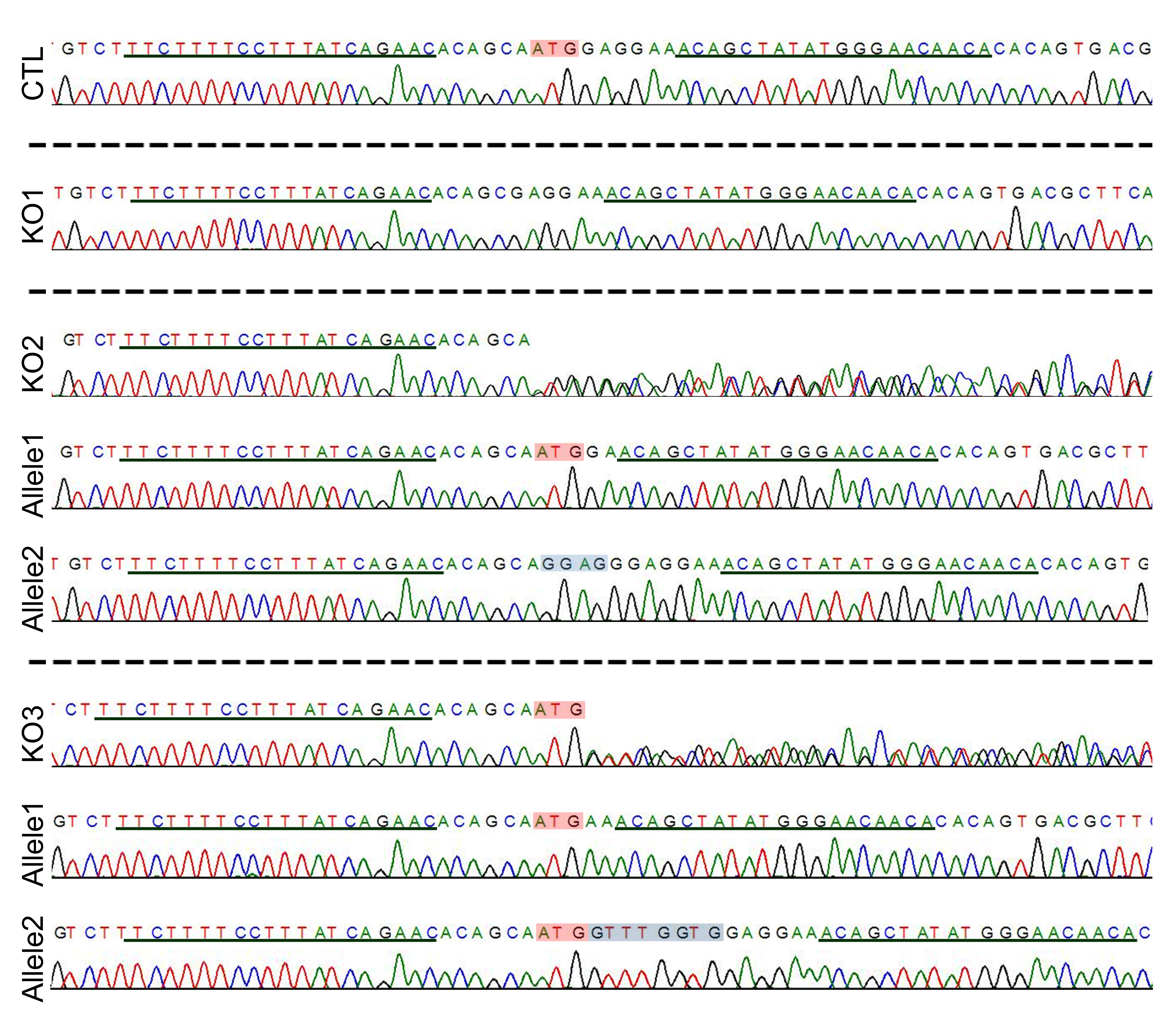

Supplement: Figure S2 — Chromatograms of sequences around TALEN targeting site in control and ZO-1 knockout clones. PCR products of TALEN targeting sites from control and ZO-1 knockout clones were directly subjected to DNA sequencing analysis (control, CTL; knockout, KO 1–3). Chromatograms of sequences for KO 2 and 3 clones showed mixed peak arrays, thus PCR products from KO 2 and 3 clones were cloned into a plasmid vector and subjected to sequence analysis (allele 1 and 2 for each clone). (TIF) [file pone.0104994.s002.tif]

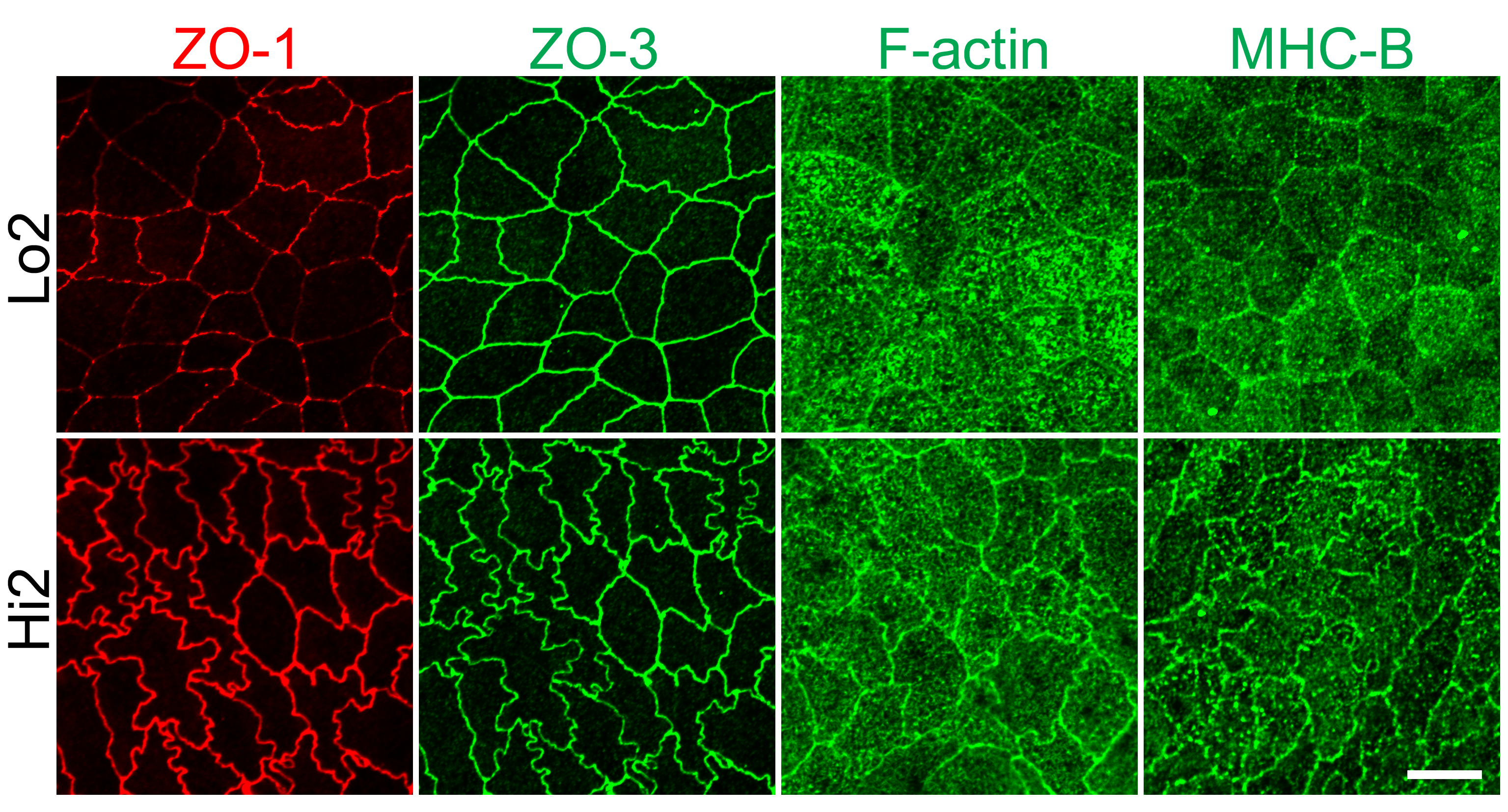

Supplement: Figure S3 — Effect of ZO-1 expression levels on the shape of cell–cell junctions and cytoskeleton in Lo 2 and Hi 2 clones. Immunofluorescence microscopic analysis of ZO-1, ZO-3, F-actin and MHC-B in Lo 2 and Hi 2 clones. (TIF) [file pone.0104994.s003.tif]

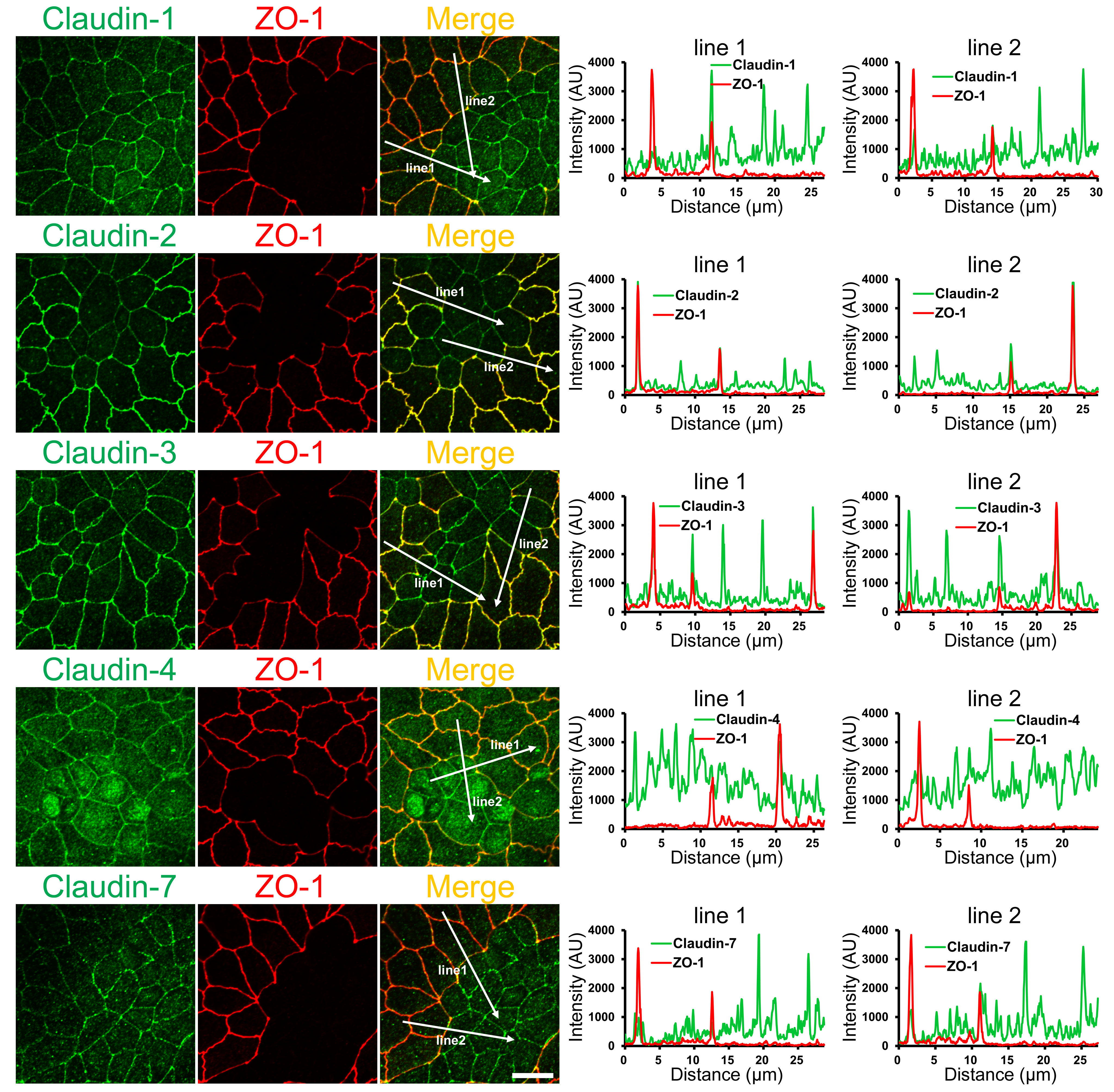

Supplement: Figure S4 — Effects of ZO-1 knockout on the localization and expression levels of claudins in ZO-1 knockout clone 3. (A) Effects of ZO-1 knockout on the localization of claudins. Control and ZO-1 knockout cells of clone 3 were co-cultured on filter inserts. Signal intensity of claudins on lines shown in confocal microscopic images (arrows) were analyzed. Claudin-2 fluorescent signal at TJs was increased but claudin-1 and -7 signals at TJs were reduced in ZO-1 knockout cells. (B) Immunoblots of claudins in control MDCK II cells and ZO-1 knockout clones. Similar expression levels of claudins were observed in control and knockout cells. (TIF) [file pone.0104994.s004.tif]
